# Mental health emergency room visits during the COVID-19 lock-down

**DOI:** 10.1016/j.pmedr.2022.102033

**Published:** 2022-10-25

**Authors:** Alexis Zebrowski, David Buckler, Giancarlo Garte, Emanuela Taioli

**Affiliations:** a Institute for Translational Epidemiology, Icahn School of Medicine at Mount Sinai, United States; b Emergency Department, Mount Sinai Health System, United States

New York City (NYC) recorded the first COVID-19 case on March 1, 2020, and soon became the epicenter of the pandemic. By March 12, 2020, a state of emergency was declared which placed the city on “pause”, included mandatory stay-at-home orders, lockdown measures, social distancing policies and a reduction of in-person work of non-essential businesses. Governor Cuomo also imposed a halt on all elective care from March 22 to June 8, 2020. Such comprehensive lockdown lead to reduction in social interaction, which in conjunction with fear of disease, economic difficulties, COVID-19 related deaths among family and friends resulted in negative effects on mental health [1]. We quantify changes in Emergency Department (ED) mental health visits in a large metropolitan health system, Mount Sinai, in NYC, which includes 3,360 beds among its seven hospitals, 136,528 average inpatient admissions, and 500,901 average ED visits.

We obtained the aggregate number of ED visits from January 1, 2018 to June 1, 2022, classified according to the ICD-10 mental health billable diagnosis codes. Compared to 2018 and 2019, there was an overall increase in ED visits for mental health in 2020 and 2021 (N = 9033 in 2018, 10,329 in 2019; 10,587 in 2020; 14,715 in 2021). The monthly analysis shows a rapid decrease in visits between March and June 2020, during the “pause” in elective care, in comparison to the same months of the previous year (−25 % roughly), and a subsequent increase over prepandemic values, which persisted in 2021 and the first part of 2022 (Fig. 1a). When the monthly mental health ED visits were calculated as a percent of total monthly ED visits, there was a significant increase in the proportion of mental health visits starting in March 2020, from 2.2 % in March to 4.1 % in May 2020; such high proportions remained stable afterward until March 2022 (Fig. 1b).

**Fig. 1:**
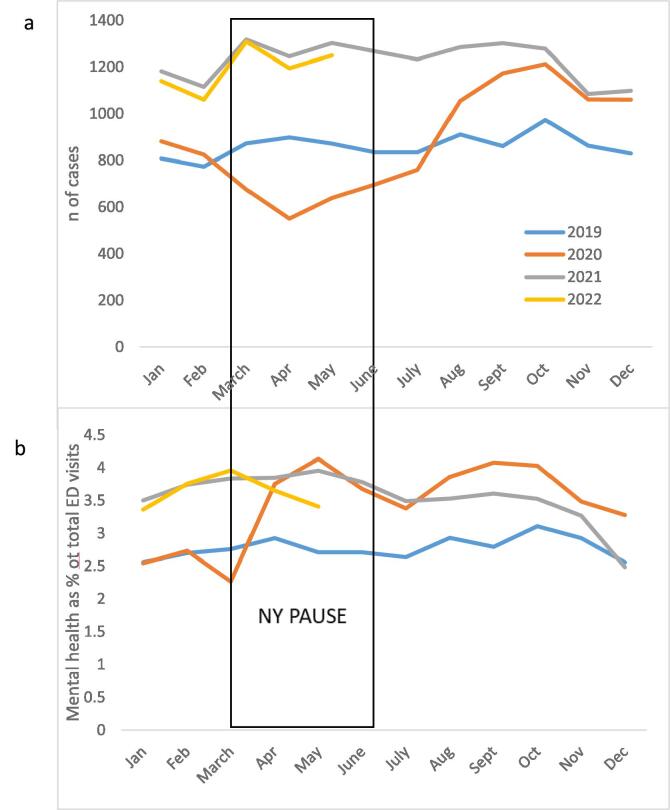
Absolute number of monthly mental health ED visits (ICD-10 diagnoses: F01-F25; F.28-F34; F39-F45; F48; F50-F53; F59-F60; F63-F66; F68, F80, F84, F88, F89-F91; F93-F95; F98; R45.7) (a); monthly number of mental health visits/total ED visits (b).

We quantified the long-term effects of the lock down on mental health emergency visits, and showed a substantial increase in mental health visits that persisted two years after the acute pandemic phase. This is an underestimate of the actual mental health burden associated with COVID-19, since it comprises the most serious cases that required an ED visit. Appropriate resources continue to be needed to address the problem.

## Declarations

The Mount Sinai IRB defined this project EXEMPT, the authors have no competing interests. There is no funding for this project. ET, AZ conceptualized and wrote the study; GG analyzed the data; DB extracted the data and operated quality control.

The dataset is available upon request to the corresponding author.

## Declaration of Competing Interest

The authors declare that they have no known competing financial interests or personal relationships that could have appeared to influence the work reported in this paper.

## Data Availability

Data will be made available on request.
